# Nonfamilial, *MPL* S505N-Mutated Essential Thrombocythaemia

**DOI:** 10.1155/2013/729327

**Published:** 2013-07-18

**Authors:** Ruth Morrell, Stephen E. Langabeer, Liam Smyth, Meegahage Perera, Gerard Crotty

**Affiliations:** ^1^Department of Haematology, Midland Regional Hospital, Tullamore, Ireland; ^2^Cancer Molecular Diagnostics, St. James's Hospital, Central Pathology Laboratory, Dublin 8, Ireland

## Abstract

Mutations of *MPL* are present in a significant proportion of patients with the myeloproliferative neoplasms (MPN), primary myelofibrosis (PMF), and essential thrombocythaemia (ET). The most frequent of these mutations, W515L and W515K, occur in exon 10 of *MPL*, which encodes the receptor for thrombopoietin. Another exon 10 mutation, *MPL* S505N, has been shown to be a founder mutation in several pedigrees with familial thrombocythaemia where it is associated with a high thrombotic risk, splenomegaly and progression to bone marrow fibrosis. Rare cases of sporadic, nonfamilial, *MPL* S505N MPN have been documented, but the presenting laboratory and clinical features have not been described in detail. The diagnosis and clinical course of a case of *MPL* S505N-positive MPN are presented with diagnostic features and treatment response resembling typical ET but with evidence of increasing bone marrow fibrosis. Further MPN cases possessing this genotype require reporting in order to ascertain whether any particular morphological or clinical features, if present, determine clinical course and aid the refinement of therapeutic options.

## 1. Introduction

Essential thrombocythaemia (ET) is a chronic myeloproliferative neoplasm (MPN) characterised by a sustained peripheral blood thrombocytosis, increased large megakaryocytes in the bone marrow and clinically by episodes of thrombosis or haemorrhage. While approximately half of all ET and primary myelofibrosis (PMF) patients harbour the *JAK2* V617F mutation, a further subset has evidence of somatic, activating, *MPL* (myeloproliferative leukemia virus oncogene) exon 10 mutations that are more frequently found in PMF than ET. *MPL* encodes the thrombopoietin receptor which is a major regulator of megakaryocytopoiesis and platelet formation. The most commonly observed mutations of *MPL* are the W515L and W515K [1, 2] although several others have been described within this codon and elsewhere within *MPL* exon 10, albeit at a much lower frequency [3–5]. ET patients harbouring *MPL* W515L/W515K mutations tend to have lower haemoglobin and higher platelet levels than those ET patients with the *JAK2* V617F, but the presence of a *MPL* W515L/K mutation does not appear to affect survival, fibrotic or leukaemic transformation [6]. Another *MPL* exon 10 activating mutation, S505N, has been described in familial thrombocythaemia [7, 8] where conversely, its presence is associated with splenomegaly, higher thrombotic risk, and progression to myelofibrosis [9]. Very few cases of nonfamilial ET with the *MPL* S505N mutation have been reported to date with little or no descriptions of clinical or laboratory features [2, 3, 5, 10]. Whether nonfamilial *MPL* S505N-positive ET is a distinct clinicopathological entity is largely unknown as these patients are often grouped with those harbouring other *MPL* W515 mutations when determining clinical correlations. A case of nonfamilial *MPL* S505N-positive ET is described in order to ascertain any distinct features associated with this genotype.

## 2. Case Report

**Figure 1:**
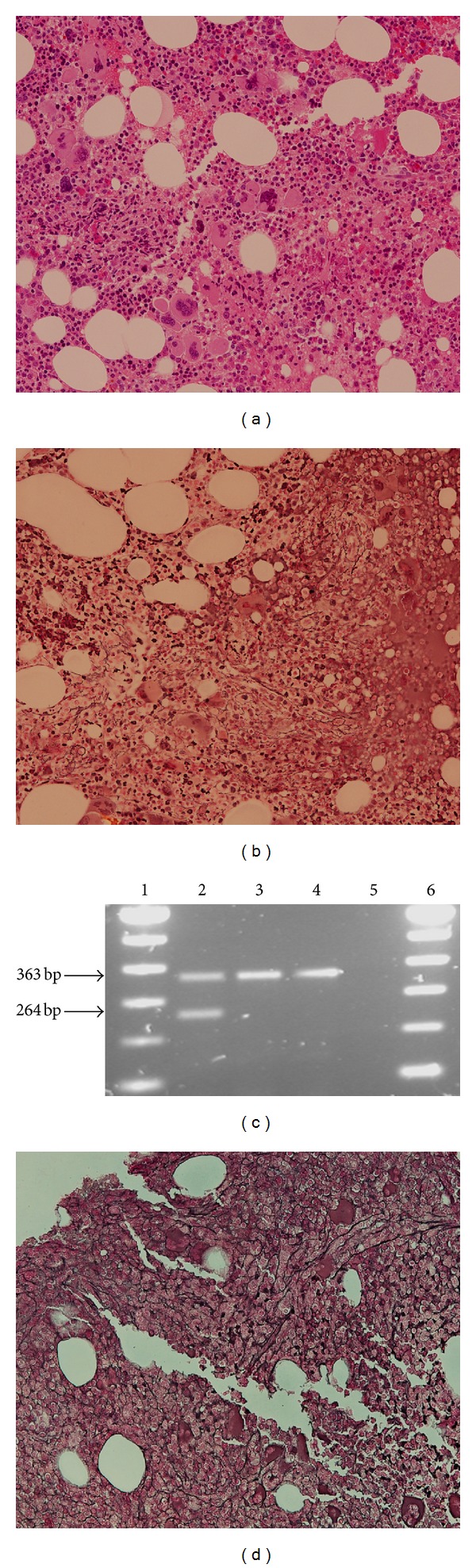
(a) Clustering of numerous pleomorphic megakaryocytes; (b) low grade bone marrow reticulin fibrosis; (c) allele-specific PCR for *MPL* S505N demonstrating wild type band (363 bp) and S505N specific band (264 bp); lanes 1 & 6, 100 bp ladder; lane 2, peripheral blood; lane 3, buccal scrape; lane 4, isolated T-cells; lane 5, water control; (d) significant increase in bone marrow reticulin fibrosis two years after presentation.

 A 55-year-old female was referred by orthopaedics who identified thrombocytosis on routine preoperative assessment for a malunioned scaphoid fracture. Her full blood count demonstrated a white cell count of 4.7 × 10^9^/L (normal range 4.0–11.0 × 10^9^/L), haemoglobin of 11.6 g/dL (normal range 11.5–16.4 g/dL), and platelets of 1188 × 10^9^/L (normal range 140–450 × 10^9^/L). The blood film appeared normal except for the thrombocytosis. There was no explanation for her thrombocytosis on history or physical examination nor was there a family history of an MPN or any thrombotic/haemorrhagic events. No evidence of splenomegaly was found on physical examination and ultrasound. LDH was 622 IU/L (normal range 240–480 IU/L) at presentation with normal inflammatory markers and a serum ferritin of 29.9 *μ*g/L (normal range 23–393 *μ*g/L). A bone marrow aspirate and trephine biopsy were performed which demonstrated a slightly hypercellular marrow in which erythropoiesis and granulopoiesis were normal, with increased numbers of eosinophils and eosinophil precursors. Megakaryocytes were increased in number with pleomorphic forms, varying from large hyperlobulated to small hypolobulated forms with some loose clustering evident (Figure 1(a)). Reticulin fibres were slightly increased with a grade 1 pattern (Figure 1(b)). Allele-specific PCR [11] did not demonstrate the presence of the *JAK2* V617F or *MPL* W515L/K mutations but detected the *MPL* S505N mutation in DNA isolated from unfractionated peripheral blood. The *MPL* S505N was not detected in DNA from a buccal scrape or isolated peripheral blood CD3+ T-lymphocytes (99.6% purity) (Figure 1(c)) indicating that the mutation was somatically acquired and not of germ-line origin. Conventional Sanger sequencing of *MPL* exon 10 was unable to demonstrate the mutation. The patient commenced on pegylated interferon at a dose of 50 *μ*g weekly, titrated to 150 *μ*g weekly over a three-month period. Her platelet count returned to normal within six months of initiating therapy and two years and presentation remains under control (476 × 10^9^/L). A subsequent bone marrow aspirate and biopsy two years from initial diagnosis showed a significant increase in the degree of fibrosis to grade 2/3 (Figure 1(d)) with no splenomegaly and no thrombotic events to date.

## 3. Discussion

One of the most frequently mutated genes in PMF and ET, apart from *JAK2*, is *MPL*, particularly at codon W515 within exon 10 [3–5]. Other infrequent mutations of *MPL* have been documented, but their clinical significance remains unclear [12–14]. The *MPL* S505N has been shown to be a founder mutation in several kindreds with familial thrombocythaemia with affected individuals harbouring this mutation having an increased risk of thrombosis and myelofibrotic transformation but do not appear to have an increased risk of leukaemic transformation [9]. Whether *MPL* S505N is a primary pathogenetic event in MPN remains to be elucidated: *in vitro* studies have shown that this mutation could not induce spontaneous cell growth, tumorigenesis, or spontaneous activation of the main receptor signal transduction pathways [3] whereas computational simulation of a structural model of *MPL* S505N supports the theory that this mutation can result in constitutive activation of the MPL-JAK2-STAT signalling pathway [15]. As the S505N mutation has been reported in both ET and PMF, further events are most likely required to influence the MPN phenotype.

As no family history of an MPN or thrombotic events was evident, the absence of the *MPL* S505N in both buccal scrape and isolated peripheral T-cells indicates that this mutation was acquired and not hereditary. Further confirmation of the somatic nature of this mutation derives from the evidence that the mutation was only detectable by allele-specific PCR indicating a mutant allele burden below the sensitivity of detection by conventional Sanger sequencing, that is, a level lower than that expected if a heterozygous, constitutional mutation was present in all cells. *MPL* S505N allele burdens, assessed by pyrosequencing, are variable [2] with levels of other *MPL* mutations, such as the W515L, detected as low as 1.5% [16]. Sensitive methodologies such as allele-specific PCR (qualitative or quantitative) or high resolution melting curve analysis are therefore required to identify all potential pathological mutations within *MPL* exon 10 and incorporated into molecular diagnostic algorithms for *JAK2* V617F-negative MPN [17].

The case described herein possesses many features of typical ET although a previous study [6] reported that ET patients harbouring *MPL* W515K mutations tend to present with a reduced bone marrow cellularity and increased megakaryocyte clustering, not obviously apparent in this case. Whether this case of sporadic *MPL* S505N ET resembles that of the familial disease harbouring this mutation remains to be seen. Close monitoring is planned with further surveillance BM biopsies required to assess levels of reticulin, an important indicator of myelofibrotic transformation in ET [18, 19].

As some small-molecule JAK2 inhibitors have shown efficacy in murine models of *MPL *W515L [20, 21], identification and characterisation of further cases of non-familial, *MPL* S505N-mutated MPN is required as these patients may benefit therapeutically from such strategies and also to determine features that may influence the risk of both thrombotic events and fibrotic disease progression.
